# Bilateral cleft lip, alveolus, and palate deformity in an infant

**DOI:** 10.11604/pamj.2022.42.316.33040

**Published:** 2022-08-29

**Authors:** Sharayu Vinod Nimonkar, Vikram Murlidhar Belkhode

**Affiliations:** 1 Prosthodontics, Crown and Bridge, Reader, Sharad Pawar Dental College and Hospital, Datta Meghe Institute of Medical Sciences, Deemed to be University, Sawangi, Wardha, Maharashtra, India,; 2 Prosthodontics, Crown and Bridge, Private Practitioner, Wardha, Maharashtra, India

**Keywords:** Bilateral cleft lip and palate, presurgical nasoalveolar molding, natal teeth

## Image in medicine

A 20-day-old infant reported with his parents a complaint of an opening over the lip and trouble in feeding. The weight of the baby at birth was 2.8 kg and medical history was not remarkable. Clinical examination manifested bilateral cleft lip, alveolus, and palate deformity. A single natal tooth in the upper anterior segment was present. The nasal rim was collapsed and the premaxilla was deviated and rotated to the left side. The alveolar segments were underdeveloped. Extraction of the natal teeth was advised followed by pre-surgical nasoalveolar molding for approximation of the lips, alveolus, and palate.

**Figure 1:**
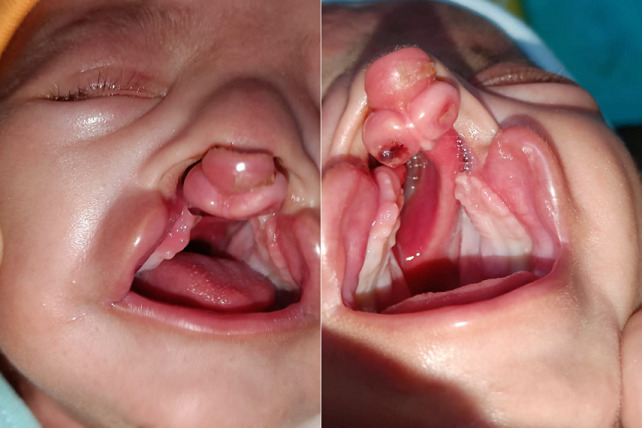
cleft lip palate and alveolus

